# Cleaving of PMMA Microstructured Polymer Optical Fibers with 3- and 4-Ring Hexagonal Cladding Structures

**DOI:** 10.3390/polym13091366

**Published:** 2021-04-22

**Authors:** Rubén Guijarro, Alberto Tapetado, David Sánchez Montero, Carmen Vázquez

**Affiliations:** Electronics Technology Department, University Carlos III of Madrid, 28911 Leganés, Madrid, Spain; ruben.guies@gmail.com (R.G.); atapetad@ing.uc3m.es (A.T.); cvazquez@ing.uc3m.es (C.V.)

**Keywords:** microstructured polymer optical fiber (mPOF), fiber cleaving, mPOF connectorization, mPOF handling, mPOFs for sensing

## Abstract

The cleaving of a novel microstructured polymer optical fiber (mPOF) to obtain an acceptable connectorized fiber end-face is studied. The effect of the blade temperature and the speed of the cutting blade on the end-face is qualitatively assessed. Recently manufactured mPOFs with air-structured 3- and 4-ring hexagonal-like hole cladding structures with outer fiber diameters of around 250 μm are employed. Good quality end-faces can be obtained by cleaving mPOF fibers at room temperature for blade temperatures within the range 60–80 °C and at a low blade speed at 0.5 mm/s. The importance of the blade surface quality is also addressed, being a critical condition for obtaining satisfactory mPOF end-faces after cleaving. From our experiments, up to four fiber cuts with the same razor blade and blade surface can be carried out with acceptable and similar fiber end-face results.

## 1. Introduction

In the last few years, polymer optical fibers (POFs) have been demonstrated as a realistic alternative to silica optical fiber (SOF) in many application fields, driven by the excellent mechanical and optical properties of the polymers. Most of the POFs in the market are composed of polymethylmethacrylate (PMMA), a material with significant interest due to its flexibility, easy handling and low cost, or other specific characteristics such as its low Young’s modulus, high thermo-optic coefficient, biological compatibility [1] and electromagnetic immunity, among others [2]. These advantages are interesting in general applications but still more in sensing applications, especially the Young’s modulus, in which large deformation can be applied without breaking the optical fiber [3], and the thermo-optic coefficient [4], which allows one to modulate the sensor response with the temperature, among other novelty applications [5].

In the beginning, POF development was closely linked to short-haul communication links due to its good properties for low-cost data transmission. These developments were mainly focused in step-index POF (SI-POF) or graded-index POF (GI-POF). Since then, other POFs have been designed and developed with other kinds of structures and polymers which provide new transmission features. One of them is the microstructure POF (mPOF). This new type of POF is likely to be of great importance because all disadvantages of conventional POF are addressed using mPOF. The light guiding mode in mPOF is different from their conventional POF counterparts. The light was guided along the length of the fiber through microscopic air channels. Modelling the pattern of these air channels allows one to develop single-mode guidance in a relatively large core. In addition, taking advantage of the intrinsically photosensitive property of the polymer and the special guidance mode of mPOF allow one to inscribe Bragg grating in POF [6], reducing the complex process of doping in polymers and opening the path for new applications [7]. On the other hand, despite the fact that PMMA is the most used polymer to manufacture mPOF, other polymers are also drawn to provide special properties to the fibers. Interesting types of polymers are the cyclic olefin copolymers (TOPAS) [8], which is regarded as the best choice for humidity insensitive fiber-optic sensors (FOS), the cyclo-olefin homopolymer (ZEONEX), which not only is humidity insensitive but also has a high glass transition temperature [9], polycarbonate (PC), with excellent clarity and impact strength properties [10], and finally, fluorinated polymer (CYTOP), which presents the advantage of longer operational distances such as its low-loss at the 1550 nm region [11].

Historically, an important disadvantage of using mPOF FOS has been its lack of connectivity with the world away from controlled laboratory environments. It is well known that the end surface quality of POFs, and more relevant in mPOF, is one of the main challengers to optimize the launch conditions. Low insertion losses and return losses are achieved with a high-quality fiber end surface. Achieving low insertion losses during the connectorization of mPOF sensors allows researchers to make the use of these sensors more widespread in many applications [12].

In this sense, many authors have been working to bridge the gap between both worlds, developing different techniques, for example: the semiconductor dicing saw and ultraviolet laser cleaving [13], or low-temperature cleaving [14]. The main drawback of these techniques is the need of expensive and complex laboratory equipment, limiting their uses only to laboratory testing. Additionally, these techniques only demonstrate their effectiveness for mPOF with fiber diameters higher than 400 μm, reducing their uses only for custom-made fibers. On the contrary, other authors have shown that a good quality end surface can also be obtained with simpler and cheaper techniques, for instance: a simple cut by razor blade [15] or a connectorization [16,17]. Among these techniques, the most extended is hot razor blade cleaving because of its high speed and effectiveness combined to the lower price per cleaving. However, in this procedure, different cutting parameters are considered to achieve a good cleaving, for instance: temperature, velocity, angle, and quality of the razor blade, as well as the temperature of the plate where the fiber is cleaved. These parameters shall be characterized for each kind of fiber because the drawing conditions and the based material play important roles in this process.

The connectorization process is one of the most promising and optimized methods for the end face termination of mPOFs. With this method, the mPOF is put into the ferrule of a connector and glued afterwards. Then, the excess fiber on the ferrule surface is cleaved using a razor blade. Finally, the end face termination is polished using a handheld tool and sandpapers with different grits. One of the major disadvantages of this process is the effectiveness of the polish stage. As a handcrafted process, the rate of failure after applying the polish stage is too high. It is due to the high temperatures generated between the fiber end and the sandpaper that they can damage the internal structures. In addition, the small particles during the polishing may block those structures. The result is an increment of the insertion losses, which are critical in mPOFs. Another issue shown is that the fiber core concentricity is not perfectly alight to the longitudinal axis of the ferrule. The concentricity of the fiber core plays a relevant role for mPOFs, since current manufacturing processes do not follow strict quality standards, leading to changes in concentricity and even in the dimensions of the structures along the fiber length. Previous analysis shows that up to yet, there is not yet a simple and effective method to end mPOF fibers with easy-to-use connectors.

This paper reports on a detailed examination of cleaving conditions in novel mPOF fibers fabricated elsewhere in PMMA material and air-structured 3- and 4-ring hexagonal-like hole arrangements with an outer fiber diameter of around 250 μm. However, the technique presented in this manuscript is also applicable for cutting different fiber materials and microstructures used in applications such as intensity fiber optic sensors or FBG sensors.

The manuscript is organized as follows. In Section 2, we describe the experimental setup developed for an automated mPOF fiber cleaving. In Section 3, we firstly introduce the mPOF fibers tested and show the experimental results obtained. We also analyze the best conditions to obtain good quality fiber end-faces after cleaving. Parameters such as blade temperature, cutting speed and blade surface status impact are addressed. In Section 4 we summarize the results obtained and compare them with previous works reported in the field of mPOF cleaving. Finally, Section 4 concludes the paper.

## 2. Experimental Setup

The proposed setup is based on a modified 3D printer structure, where the printer nozzle was removed, to explore different cleaving configurations in order to achieve a repeatable, precise and flexible method to cut different types of mPOF reducing the connectorizing optical losses. The developed cleaving machine performs cuts at a configurable temperature and speed, in contrast to commercially available hot-knife solutions for the same purpose, and shares the same concepts for POFs such as being low-cost and do-it-yourself. The structure of this device consists of 3 displacement axes actuated by NEMA 17 stepper motors. The printing bed and the razor blade holder are connected to the X and Y axis motor, respectively, through a belt system, allowing a maximum accuracy of 0.2 mm. The cutting direction is defined by the Z axis, corresponding to the vertical movement based on a double spindle motor. The Z axis is the highest accuracy one compared to the other belt-based movement axes. The minimum possible displacement to be performed in Z is 6.25 µm within a speed range between 0.2 mm/s and 5.6 mm/s, achieving the recommended values in the previous literature to prevent the formation of cracks in the cut surface [15,18,19,20]. All stepper motors are driven by a bipolar stepper motor driver; this driver can enhance the accuracy of the motors using microstepping control techniques, improving this magnitude up to 32 times.

**Figure 1:**
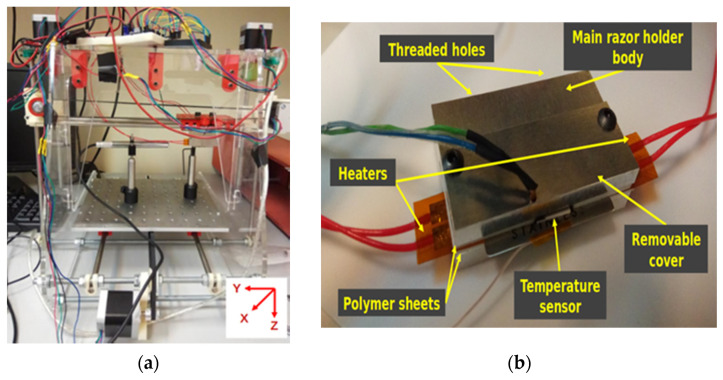
(**a**) Picture of the mPOF cleaving machine; (**b**) detailed view of the razor blade holder and its parts.

Several structural modifications have been included in the 3D printer structure to adapt it for mPOF cutting purposes, as is shown in Figure 1a.

In this figure, an aluminum table replaces the printing bed. This mechanical part presents M6 threaded holes distributed as a standard optical table. The space between holes is 25 mm in one direction and 22.5 mm in the other. This design provides a great flexibility grade, allowing one to use standard optical holders and posts to place the required components in the system.

In order to cleave the mPOF, it is required to position the razor blade as close as possible to the fiber avoiding the contact with the ferrule; for that, a USB microscope is attached to the structure using optical posts and holders. The microscope is placed perpendicular to the blade and the fiber connector to obtain a good view of the position of both prior to running the automatic cleaving procedure. The fiber connector is attached to the optical table through a ferrule holder, which can hold ferrule diameters up to 2.5 mm, typical of small form factor connectors and other standard connectors such as FC connectors to be used in this work [21]. This retention device is placed in the optical table using a system of optical posts with a similar height as the one utilized for the microscope, easing the image focus process.

The holder displayed in Figure 1b securely grips the razor in the Y axis carrier during the whole of the cleaving process. This aluminum structure consists of a fixed part with threaded holes to attach it to the carrier, and a removable part which presses the interior components. The blade is packed in the middle of the holder structure between a pair of flexible heaters, aiming to keep both sides of the razor at the same temperature. The dimensions of the heater are 10x50 mm with a heating power of 5 W/inch, providing a maximum temperature of 200 °C. To reduce the thermal losses, the contact layer of the metallic holder with the resistors is made of a thermal insulation polymer. The melting temperature of this polymer is above 300 °C with a low thermal conductivity of 0.25 W/(m⋅K) measured at 23 °C. Moreover, a tiny resistance temperature resistor, PT100, is included to have feedback information of the razor temperature. The dimensions of the PT100, with a nominal resistance of 100 Ω, are 2 × 10 mm, making it easy to integrate in the system. The temperature range of this sensor goes from −50 to 500 °C, with a maximum thermal response of 0.1 s.

**Figure 2:**
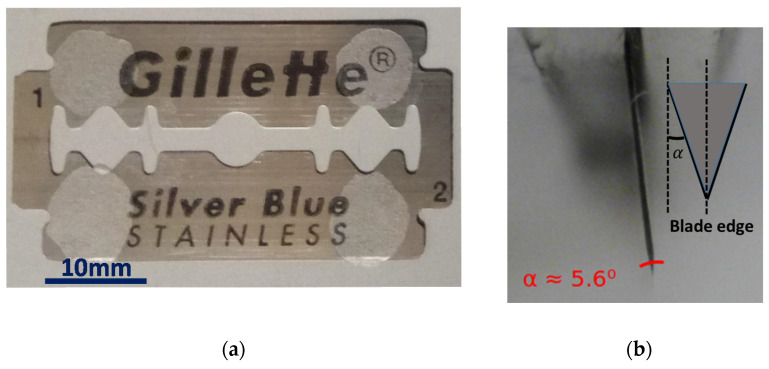
(**a**) Razor blade used for cleaving; (**b**) detailed view of the razor blade angle.

The blade used for the cleaving tests is a shaving double razor blade (see Figure 2a). The blade edge presents an angle approximately of 5.6°, as shown in Figure 2b. It is required to remove the remaining adhesive attached to the blade before being used. The method used to remove the adhesive and the edge angled shape will be determinant factors of the mPOF cleaving process results.

To ease the use of the cleaving device, a graphic interface has been developed in National Instruments LabVIEW. This software allows one to integrate in the same user interface the control of all actuators, the temperature sensors and a real-time image of the cleaving process from the microscope. The temperature of the razor edge is managed by a PID controller which provides a stable temperature with an error of 0.2 °C due to the resolution of the microcontroller analogue-to-digital converter (ADC) and the characteristics of the selected reading circuit. An Arduino Mega 2560 board has been selected as a microcontroller to command the system features because of its ease of integration with the programming software.

**Figure 3:**
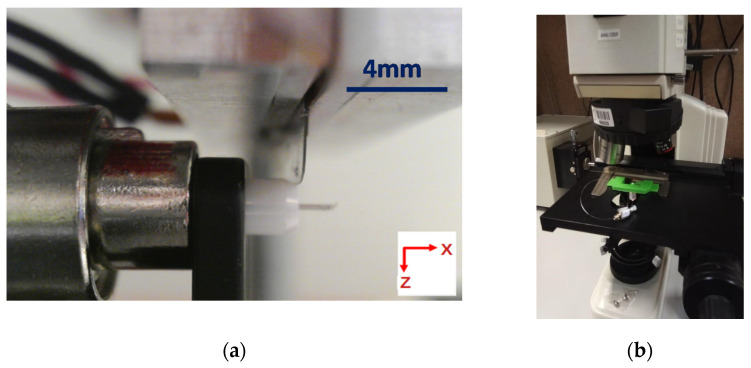
(**a**) Picture of the cleaver before stating the cleaving sequence. (**b**) Optical microscope with ferrule holder for characterizing the cleave.

In Figure 3a, the image recorded in the USB microscope to position the razor in relation to the fiber before starting the cleaving sequence can be observed. Once the blade is positioned and the temperature is stable in its edge, the next steps in the cleaving sequence consist of a descending movement of the blade in Z direction, followed by a movement of the ferrule in X direction to separate the fiber surface from the hot blade.

To evaluate the quality of the cleaving process, an additional optical microscope has been used. The selected optical objectives are 50× to obtain detailed images of the mPOF surface after the cleaving process. In addition to the microscope, a ferrule holder has been designed to place the fiber connectors in the stage clips in a steady position facing the microscope lenses. The microscope and the holder can be observed in Figure 3b. Moreover, a digital camera model is connected to the microscope to display the received information from the objectives in a computer.

## 3. Experimental Results

This section reports on the detailed examination and experimental results of mPOF cleaving and its connectorization employing the automated apparatus described in Section 2. Results on the effects of mainly blade temperature, blade speed and blade surface quality on the cutting of such a fiber are presented. 

**Figure 4:**
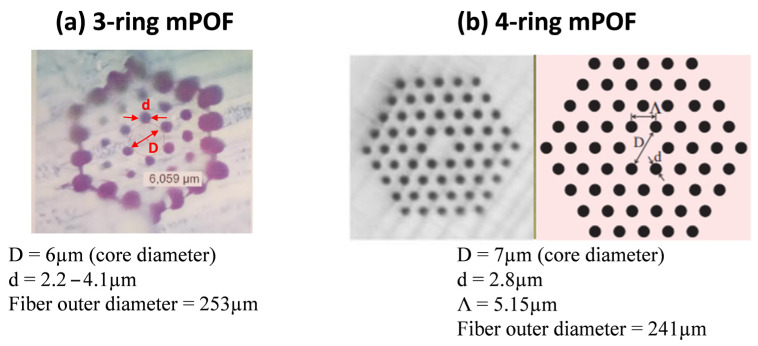
(**a**) Microscope image of the 3-ring hexagonal air hole distribution mPOF. (**b**) Microscope image and idealized target geometry for the 4-ring hexagonal air hole distribution mPOF. mPOF structural dimensions are provided.

The mPOF fibers studied here have 3- and 4- ring-like hole arrangements, thus allowing single-mode POF operation [22]. They were fabricated from amorphous polymethylmethacrylate (or PMMA) in a two-step process: on the one hand, the design and creation of a preform containing a large-scale version of the desired fiber and, on the other, the precise heating and drawing of the preform to the final fiber. The glass transition temperature of the PMMA under this study is around 110 °C. This preform was firstly annealed for 14 days at 90 °C in a climate chamber to avoid the appearance of air bubbles during drawing as a consequence of a small amount of volatile particles present in the preform [23]. The required hole pattern was next drilled in this preform cylinder of high purity PMMA. This cylinder was then drawn to a 60 mm diameter rod (primary mPOF preform) and sleeve, i.e., intermediate stretched, to a microstructured cane of around 6 mm diameter prior to the final drawing. The latter was then drawn to mPOF fibers with an outer diameter of around 250 μm and air holes of around 3 μm of diameter. Further details about the manufacturing process of the mPOF samples are reported in [24,25]. The mPOF fibers tested for optimized cleaving were single-core by drilling a central hole to the classical hexagonal ring pattern [26] in both a 3-ring and a 4-ring hexagonal air hole distribution. Structural dimensions of the mPOFs tested are shown in Figure 4.

### 3.1. mPOF Samples Preparation

**Figure 5:**
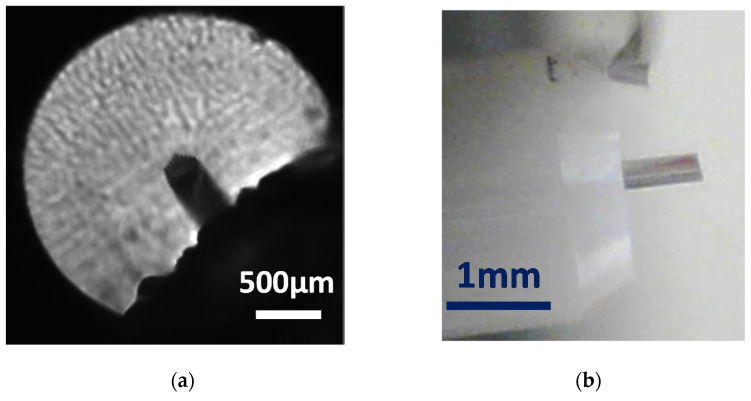
Pictures of one mPOF sample connectorized to a FC connector before automated cleaving testing. (**a**) Top view, (**b**) Lateral view.

The mPOF fiber samples were first manually cleaved with a razor blade at room temperature and then inserted into 250 μm ferrule hole diameter FC connectors compatible with the outer diameters of the mPOFs tested. mPOFs with 3-ring and 4-ring hexagonal air hole cladding structures were tested. In all samples, the mPOF fiber end protuded around 2–3 mm beyond the FC connector ceramic end prior to the automated cleaving process. Some figures of the mPOF sample before automated cleaving are shown in Figure 5. However, due to diameter tolerances in some samples, the mPOF outer diameter had to be slightly reduced by the immersion of the mPOF sample into pure acetone solvent for a few seconds. This mPOF etching process is similar to that reported in [27] with negligible impact on the mPOF mechanical and light guiding properties. It should be mentioned that during this process, epoxy was placed at the fiber end to avoid the degradation of the mPOF ring-like hexagonal hole structure due to the acetone solvent. On the other hand, in some other samples the mPOF outer diameter was less than the 250 μm, for which the ferrule was selected. This problem was addressed by inserting the mPOF fiber into a short length of polyimide tubing to increase the outer diameter and to match with the ferrule of the FC connector. The mPOF was then fixed to the FC connector by an UV-sensitive adhesive through the front of the connector. After 15 min of cure through UV radiation between 2–15 W, the mPOF sample was ready for the automated cleaving testing.

### 3.2. Impact of Blade Temperature and Blade Speed

**Figure 6:**
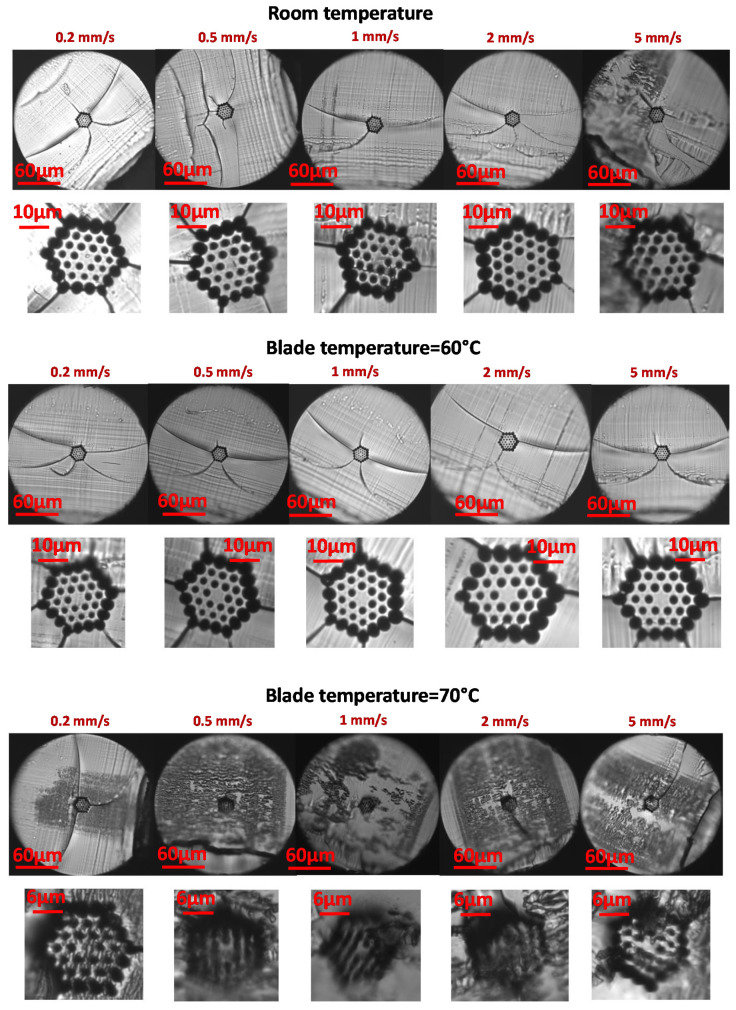

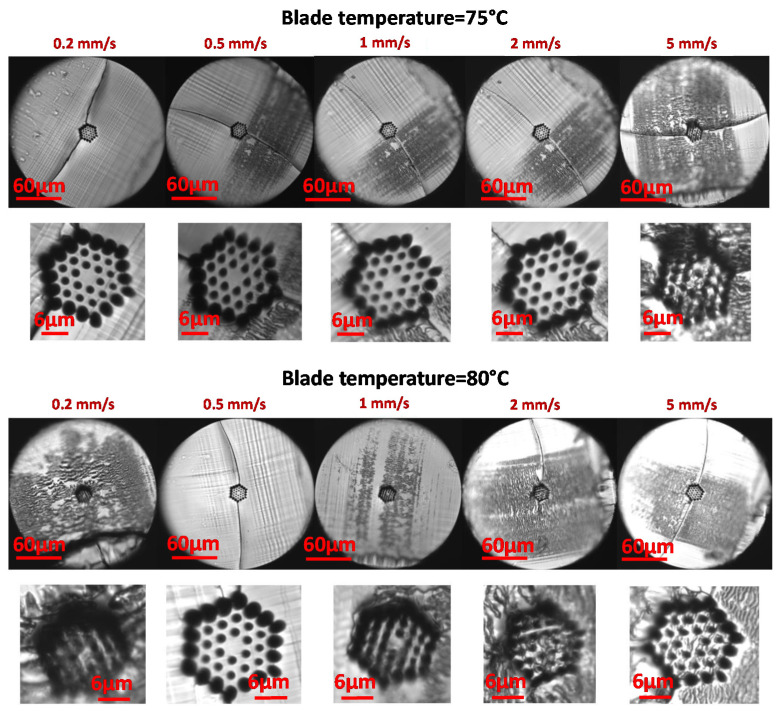
3-ring mPOF end faces at different blade temperatures and blade cutting speeds.

Different razor blades were exposed to a various combination of temperatures within the range of 50–85 °C, with increments of 5 ± 0.2 °C. This blade temperature range is similar to that proposed by other authors but for other mPOF fibers [15]. Greater blade temperature values led to air-structure degradations due to fiber melting and were not evaluated in this work. In all cases, the blade heating duration was of around 10 min before cleaving to allow very stable and precise temperature values. The fiber was at 23 °C, room temperature. In addition, different blade speeds were tested being moved while fiber cutting at speed ranging from 0.2 mm/s to 5 mm/s. Figure 6 shows some representative examples of the mPOF end faces after cleaving for different setups.

The best mPOF end-faces occur for low-speed blade cutting and for blade temperatures within 60 to 80 °C, where the surrounding hole structure is relatively intact and little evidence of surface damage is observed. For high blade speeds such as 2 or 5 mm/s, results were not satisfactory, and the high degree of damage is clearly noticed in most of the cases. Moreover, most of the fine bridges between air holes in the ring-like structure showed a fracture. In all cases, a crack propagation at the fiber center and along the whole fiber diameter is noticed due to the mPOF’s more brittle behavior at room temperature in comparison with the case of heating up the surface. This fact leads to a similar behavior to that of for a standard glassy fiber cleaving process, where the fiber is pulled axially to propagate the crack in the initial crack tip. However, the fiber core and the shape of the ring-like air-structures are maintained and the damage seems restricted to a thin layer on the surface. Cutting steps for the blade movements, i.e., step size of the motor, are also clearly observed in the form of transverse striations.

For a significant number of the end-faces, there appeared to be a fracture at the beginning of the fiber cutting process due to the initial pressure of the blade on the fiber surface as well as the more brittle behavior of the polymer material at ambient temperature. It is worth mentioning that the mPOF images depicted in Figure 6 and following were captured by a microscope stage with an objective lens, being placed the fiber center, i.e., the fiber core region, at the focal length. The blurred (defocused) zones observed outer the fiber center from the different end-face microscopic images were due to the non-focal position of the fiber end, i.e., these parts of the cleave were not in the same focal plane as the rest. This is because of the angle of the razor blade cutting surface that leads to a “diagonal” or angled cleaving to the fiber axis instead of a perpendicular one.

**Figure 7:**
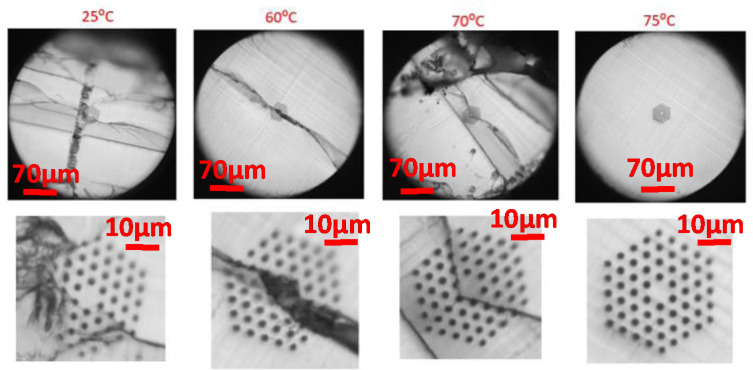
4-ring mPOF end faces at different blade temperatures and 0.5 mm/s blade cutting speed.

We also explored the feasibility of a 4-ring mPOF with hexagonal holes distribution for different blade temperatures and a cutting speed of 0.5 mm/s that showed the best fiber cleaving results for the 3-ring mPOF. Figure 7 shows some representative examples of the mPOF cleaving with this air-hole mPOF structure. As expected, good cleaving results are also obtained, although in this case, for temperatures smaller than 75 °C, a noticeable crack in the fiber end face is observed due to the ductile mPOF behavior for low blade temperature values. The mPOF fiber was at room temperature.

**Figure 8:**
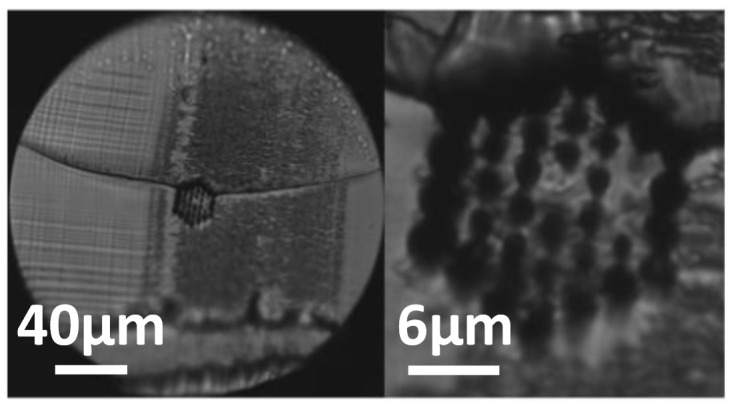
mPOF fiber end unsatisfactory surface roughness due to bad blade condition.

Finally, it is worth pointing out that some of the mPOF cross-sections analyzed after cleaving showed a grey zone, indicative of a slightly rougher surface causing scattering of the reflected light. This is due to the blade surface quality, a critical condition for mPOF cleaving that will be addressed in the next section. A detailed picture of this effect is shown in Figure 8. It can also be seen in some fiber end images within Figure 6 and Figure 7, respectively.

### 3.3. Impact of Blade Condition

**Figure 9:**
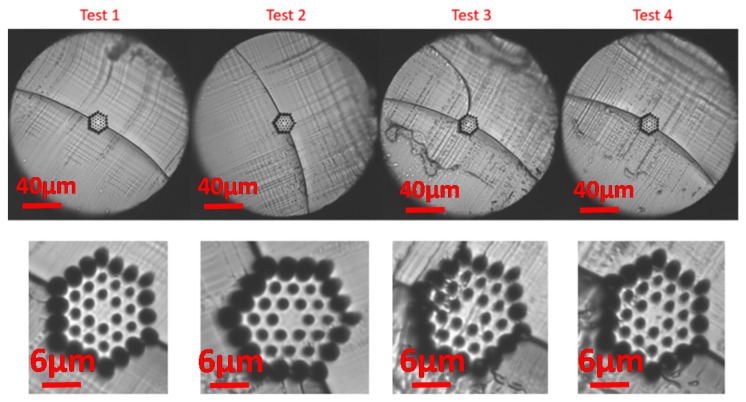
mPOF fiber ends using the same blade surface. Up to four fiber cleavings with satisfactory results can be obtained. Blade temperature was 60 °C at 0.5 mm/s of blade cutting speed.

From our experiments, we noticed that the blade condition played a key role in the quality of the fiber cleaving. To eliminate the effect of changes in blade condition caused by cutting the PMMA several times using the same blade surface, the blade was mounted on an automated screw-driven slide, thus being able to be moved before every cut. This is mainly due to the fact that the PMMA material accumulated onto the blade surface from successive fiber cuts. For the particular blade and mPOF fiber under study is was found that:

- Up to four fiber cleavings could be carried out using the same blade surface to this purpose. An example of the cleaving results obtained is depicted in Figure 9. Although the fiber surface degradation is more noticeable (more cracks, dirt and roughness) while increasing the number of cuts, this fact essentially does not affect the light transmission capacity at the fiber end-face as the ring structure is being maintained (see bottom figures within Figure 9), thus the light is still guided within the mPOF fiber core. This number of fiber cuts is a pessimistic upper bound value from our experiments and trials. Additional blade uses for fiber cleaving make it impossible to produce clean end-faces without additional blade treatment. Consequently, the mPOF fiber should slightly be moved to another blade position for a new fiber cut.
- By immersing the blade into acetone for around half an hour and after drying blade, the blade could be reused with satisfactory mPOF cleaving results.
- There was no noticeable dependency of the blade temperature, blade speed and/or mPOF fiber type on the number of satisfactory fiber cleavings using the same blade surface.

**Figure 10:**
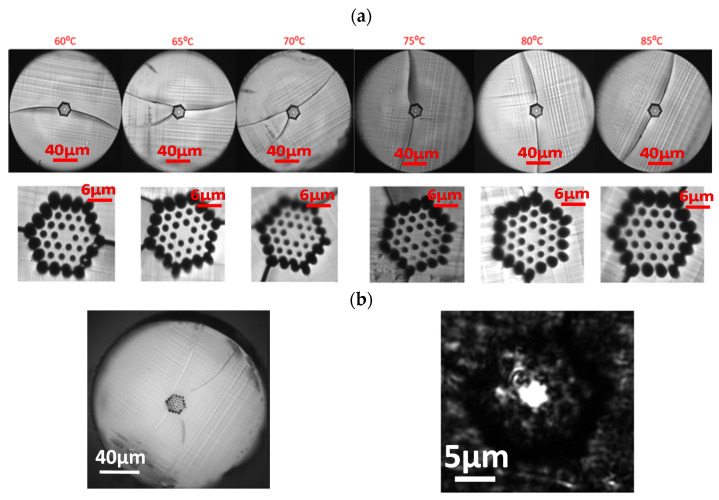
(**a**) 3-ring mPOF end faces at different blade temperatures from 60 °C to 85 °C, at a blade cutting speed of 0.5 mm/s after blade treatment. (**b**) Ouptut light pattern obtained for a 3-ring mPOF after fiber cleaving.

Figure 10a shows some resulting 3-ring mPOF fiber cleavings after half an hour acetone immersion blade treatment after each fiber cut. In all cases, the results were satisfactory at the first fiber cleaving trial. Figure 10b shows a microscope picture of the output light distribution achieved at the fiber end after fiber cleaving that shows how the light is confined to the core region.

**Figure 11:**
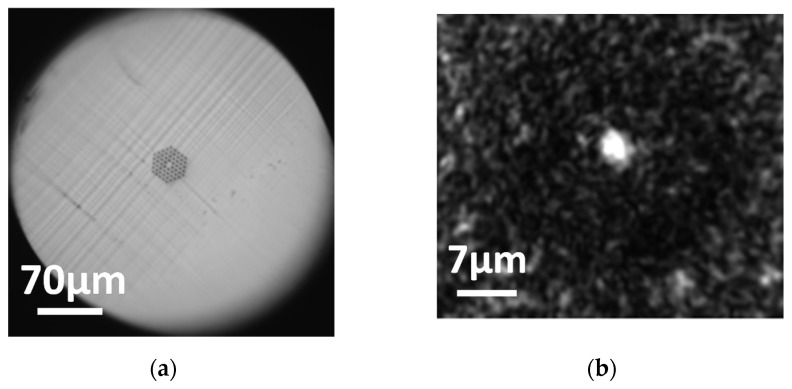
(**a**) 4-ring mPOF surface after blade treatment and cleaving. (**b**) Ouptut light pattern obtained. Blade temperature was 75 °C at a blade cutting speed of 0.5 mm/s.

Similar blade condition analysis and tests were carried out for the 4-ring hexagonal air hole distribution mPOF, thus leading to similar conclusions with respect to the 3-ring counterpart. A detailed view of the cleaving process result is provided in Figure 11, showing the good quality of the resulting output light pattern obtained at a blade temperature of 75 °C, a blade speed of 0.5 mm/s and acetone blade treatment.

## 4. Discussion

The mPOF manufacturing is not a mature technology yet, and different technologies for fiber fabrication have been reported, thus leading to customized and specialty mPOF fiber structures and materials to achieve singlemode operation in POF fibers. This means that specific parameters for cleaving novel mPOFs must be studied to determine the optimal conditions to achieve high quality fiber end-faces for further mPOF use, mainly in sensing applications [28] including the measurement of physical parameters, e.g., pressure, strain [29], torsion, temperature [30], and the detection of biomedical parameters such as refractive index and microfluidic flow rate [31]. In some cases, the connections are made using a 3D translation platform together with matching gel or by focusing the light using lenses or an objective microscope [32]. Anyhow, as previously stated, for more reliable coupling/decoupling features, the use of connectors is required.

Table 1 summarizes the optimal mPOF cleaving conditions previously reported in literature for the case of chopping the fiber with a razor blade. This table also includes the optimal cleaving conditions obtained for the mPOFs studied in this work. A total number of 101 samples of mPOF-3, i.e., a 3-ring mPOF, were cut with blade temperatures ranging from 50 °C to 85 °C and blade speeds from 0.2 mm/s to 5 mm/s. For a mPOF-4, i.e., a 4-ring mPOF, 20 samples were cut ranging from 70–75 °C for a blade speed of 0.5 mm/s.

In most of the mPOF cleaving methods shown within Table 1, both the blade and the fiber must be pre-heated at a temperature below the glass transition temperature to make the polymer ductile and prevent crazing, and where different polymers and outer diameters require different cleaving temperatures. In contrast, from our work we can also obtain good cleaving results for the fibers studied at ambient temperature. The equilibration time in our experiments is set to 10 min for two main reasons, although it could be reduced to few minutes. On the one hand, to precisely stabilize the blade temperature set for the experiments. On the other hand, for blade temperatures great enough, as the blade is located near to the fiber end-face there is a temperature gradient that allows the mPOF fiber near to the end-face behaving as a ductile material at its cutting surface, thus avoiding the need for fiber heating. We estimate a thermal gradient difference of around 10 °C lower from the temperature sensor readout located at the blade surface to the mPOF fiber end-face. Both the thermal gradients from the sensor’s location at the blade surface to the blade edge and from the blade edge to the fiber end-face are considered for this estimation. From the blade temperatures analyzed and tested in our study as well as this thermal gradient estimation, the expected lowest temperature values at the mPOF sample end-face are of around 50 °C. This temperature was previously addressed as the temperature for that of a ‘brittle-to ductile’ transition can occur in PMMA materials [33]. In all cases, lower cutting speeds avoid the appearance of crazing due to the temperature–time equivalence in polymers, a behavior that is addressed in [26]. In this last work, they also used a sawing motion with a blade angle between 1° and 5° to reduce the initial damage to the end-face with cutting speeds as low as 0.018 mm/s that required a high-precision motion stage, thus adding some complexity to the overall motion system.

**Table 1:** Comparison between different mPOFs and their corresponding cleaving parameters reported. The values included correspond to optimal cleaving conditions determined from authors.

POF Fiber (Outer Diameter)	Blade T (°C)	Platen/Fiber T (°C)	Cutting Speed (mm/s)	Equilibration Time ^1^	Cutting Procedure	Ref
**mPOF-3** **PMAA** (253 μm)	60–75	ambient	<0.5 mm/s	10 min	Automatic	This work
**mPOF-4** **PMMA** (241 μm)	70–75	ambient	<0.5 mm/s	10 min	Automatic	This work
**mPOF** **(PMMA)** (150 μm)	80	75–80	Not controlled	20 s	Manual	[17]
**smPOF ^2^** **PMMA** (<150 μm)	80	30–40	Not controlled	Not applied	Manual	[18]
**GI-mPOF** **PMMA** (400 μm)	50–80	85–95	0.03–7	>60 s	Manual and automatic	[19]
**mPOF** **(PMMA)** (90 μm)	ambient	ambient	0.018(sawing)	Not applied	Sawing	[34]
**mPOF** **(PMMA)** (125 μm)	77.5	77.5	5.6	20 s	Automatic	[35]
**mPOF** **(TOPAS)** (280 μm)	40	40	5.6	20 s	Automatic	[35]

^1^ Defined as the time the fiber is being heated before cut; ^2^ smPOF: single-mode POF.

The optimal blade temperatures for the mPOFs used in this work are similar to that of those reported for other mPOF types with similar PMMA material. However, the slight temperature range differences are mainly due to the different outer diameters of the mPOF fibers analyzed. In contrast, humidity insensitive polymer TOPAS (grade 8007) material [27] exhibits a different optimal temperature. While not conclusive, none of the previous works estimate the number of fiber cuts that can be carried out with satisfactory and similar cleaving results using the same blade surface. It should be noted that the estimation provided within this work (up to four cleavings) is restricted to the mPOF fibers studied wherein as well as to the razor blade employed in the experiments. However, we believe this result could be extended to other blades employed when cleaving and to other PMMA-based mPOF fibers with different n-ring hexagonal cladding structures. Due to the slight dependency of the Young’s modulus on the cladding structure as well as the close behavior of the Young’s modulus with respect to temperature for different cladding structures [36], the range of the cleaving parameter settings (blade speed and/or blade temperature ranges) reported within the manuscript could be applied to other PMMA-based mPOF fibers. However, we expect slight differences for the optimal cleaving parameters, particularly in mPOFs with a greater or more complex ring structure, although still being within the parameter setting ranges proposed. A slightly lower blade speed setting would be recommended to still guarantee that the greater number of fine bridge structures between the holes would not be destroyed. A slightly lower blade temperature would be also desirable to mitigate the temperature gradient influence of the blade on the mPOF fiber end face surface, as fibers with greater n-ring structures show a more noticeable Young’s modulus variation.

## 5. Conclusions

In this paper, we report on the optimal conditions for cleaving recently developed mPOFs with an outer diameter of around 250 μm and 3-ring and 4-ring-like hexagonal air hole structures. Good quality fiber end-faces after cleaving can be obtained with no need for fiber pre-heating, i.e., at room temperature. From our experiments and trials, special attention must be paid to the razor blade’s surface quality to prevent bad unsatisfactory cleaving results. Up to four cuts with acceptable and similar cleaving quality can be achieved with no need for blade replacement if we attend to maintaining the light guiding capacity within the fiber core at the fiber end-face of the mPOF samples tested. This number of fiber cuts is a pessimistic upper bound value from our experiments and trials. Beyond this value, another blade surface should be selected to perform a following fiber cut. Specific blade temperature and cutting speeds are also addressed for optimal cleaving results for this novel mPOF fiber manufactured. Good cleaving results were achieved without specifically heating the optical fiber surface, providing a simpler technique requiring less power consumption. We believe this study will help to bridge the gap between the fabrication and manufacturing of mPOFs and their real-field applications and future market introduction, mostly limited by the lack of reliable mPOF connectorization solutions. Planned extensions of this study include examining the resulting mPOF connectorization losses after this optimal fiber cleaving analysis.

## Data Availability

Not applicable.

## References

[1] Gierej A., Filipkowski A., Pysz D., Buczyński R., Vagenende M., Dubruel P., Berghmans F. (2020). On the Characterization of Novel Step-Index Biocompatible and Biodegradable poly (D, L-lactic acid) Based Optical Fiber. J. Lightwave Technol..

[2] Zubia J., Arrue J. (2011). Plastic Optical Fibers: An Introduction to Their Technological Processes and Applications. Opt. Fiber Technol..

[3] Pérez-Castellanos J.L., Montero D.S., Vázquez C., Zahr-Viñuela J., González M. (2016). Photo-Thermo-Mechanical Behaviour Under Quasi-Static Tensile Conditions of a PMMA-Core Optical Fibre. Strain.

[4] Tapetado A., Vázquez C., Zubia J., Arrue J. (2013). A temperature sensor based on a polymer optical fiber macro-bend. Sensors.

[5] Leal-Junior A., Avellar L., Frizera A., Marques C. (2020). Smart textiles for multimodal wearable sensing using highly stretchable multiplexed optical fiber system. Sci. Rep..

[6] Marques C.A., Bilro L.B., Alberto N.J., Webb D.J., Nogueira R.N. (2013). Narrow bandwidth Bragg gratings imprinted in polymer optical fibers for different spectral windows. Opt. Commun..

[7] Tapetado A., Montero D.S., Webb D.J., Vázquez C. (2014). A Self-Referenced Optical Intensity Sensor Network Using POFBGs for Biomedical Applications. Sensors.

[8] Emiliyanov G., Jensen J.B., Bang O., Hoiby P.E., Pedersen L.H., Kjær E.M., Lindvold L. (2007). Localized biosensing with Topas microstructured polymer optical fiber. Opt. Lett..

[9] Woyessa G., Fasano A., Markos C., Stefani A., Rasmussen H.K., Bang O. (2017). Zeonex microstructured polymer optical fiber: Fabrication friendly fibers for high temperature and humidity insensitive Bragg grating sensing. Opt. Mater. Express.

[10] Fasano A., Woyessa G., Stajanca P., Markos C., Stefani A., Nielsen K., Rasmussen H.K., Krebber K., Bang O. (2016). Fabrication and characterization of polycarbonate microstructured polymer optical fibers for high-temperature-resistant fiber Bragg grating strain sensors. Opt. Mater. Express.

[11] Leal-Junior A.G., Theodosiou A., Min R., Casas J., Díaz C.R., Dos Santos W.M., Pontes M.J., Siquiera A.A.G., Marques C., Kalli K. (2019). Quasi-distributed torque and displacement sensing on a series elastic actuator’s spring using FBG arrays inscribed in CYTOP fibers. IEEE Sens. J..

[12] Leal-Junior A.G., Frizera A., Marques C., Sánchez M.R., Botelho T.R., Segatto M.V., Pontes M.J. (2018). Polymer optical fiber strain gauge for human-robot interaction forces assessment on an active knee orthosis. Opt. Fiber Technol..

[13] Atakaramians S., Cook K., Ebendorff-Heidepriem H., Afshar S., Canning J., Abbott D., Monro T.M. (2009). Cleaving of Extremely Porous Polymer Fibers. IEEE Photonics J..

[14] Ghirghi M.V.P., Minkovich V.P., Villegas A.G. (2014). Polymer Optical Fiber Termination with Use of Liquid Nitrogen. IEEE Photonics Technol. Lett..

[15] Law S.H., van Eijkelenborg M.A., Barton G.W., Yan C., Lwin R., Gan J. (2006). Cleaved end-face quality of microstructured polymer optical fibres. Opt. Commun..

[16] Abang A., Webb D.J. (2012). Demountable connection for polymer optical fiber grating sensors. Opt. Eng..

[17] Abang A., Sáez-Rodríguez D., Nielsen K., Bang O., Webb D.J. Connectorisation of fibre Bragg grating sensors recorded in microstructured polymer optical fibre. Proceedings of the SPIE Fifth European Workshop on Optical Fibre Sensors.

[18] Abdi O., Wong K.C., Hassan T., Peters K.J., Kowalsky M.J. (2009). Cleaving of solid single mode polymer optical fiber for strain sensor applications. Opt. Commun..

[19] Law S.H., Harvey J.D., Kruhlak R.J., Song M., Wu E., Barton G.W., van Eijkelenborg M.A., Large M.C.J. (2006). Cleaving of microstructured polymer optical fibres. Opt. Commun..

[20] Law S.H., Barton G.W., van Eijkelenborg M.A., Yan C., Lwin R., Gan J. The effect of fabrication parameters on the cleaving of microstructured polymer optical fibers. Proceedings of the SPIE Novel Optical Systems Design and Optimization IX.

[21] TIA (Telecommunications Industry Association) Standard FOCIS 4 (Fiber Optic Connector Intermateability Standards) EIA/TIA-604-04, Sep. 2004. https://tiaonline.org/.

[22] Van Eijkelenborg M.A., Argyros A., Barton G., Bassett I.M., Fellew M., Henry G., Zagari J. (2003). Recent Progress in Microstructured Polymer Optical Fibre Fabrication and Characterisation. Opt. Fiber Technol..

[23] Kuzyk M.G. (2006). Polymer Fiber Optics: Materials, Physics, and Applications.

[24] Arrospide E., Durana G., Azkune M., Aldabaldetreku G., Bikandi I., Ruiz-Rubio L., Zubia J. (2018). Polymers beyond standard optical fibres- fabrication of microstructured polymer optical fibres. Polym. Int..

[25] Arrospide E., Bikandi I., Larrañaga I., Cearsolo X., Zubia J., Durana G. (2019). Harnessing Deep-Hole Drilling to Fabricate Air-Structured Polymer Optical Fibres. Polymers.

[26] Argyros A., Leon-Saval S.G., Pla J., Docherty A. (2008). Antiresonant reflection and inhibited coupling in hollow-core square lattice optical fibres. Opt. Express.

[27] Janting J., Pedersen J.K.M., Inglev R., Woyessa G., Nielsen K., Bang O. (2019). Effects of Solvent Etching on PMMA Microstructured Optical Fiber Bragg Grating. J. Lightwave Technol..

[28] Talataisong W., Ismaeel R., Beresna M., Brambilla G. (2019). Suspended-Core Microstructured Polymer Optical Fibers and Potential Applications in Sensing. Sensors.

[29] Durana G., Gomez J., Aldabaldetreku G., Zubia J., Montero A., de Ocariz I.S. (2012). Assesment of an LPG mPOF for strain sensing. IEEE Sens. J..

[30] Yue X., Chen H., Qu H., Min R., Woyessa G., Bang O., Hu X. (2020). Polycarbonate mPOF-Based Mach–Zehnder Interferometer for Temperature and Strain Measurement. Sensors.

[31] Liu Z., Tam H., Htein L., Tse M.V., Lu C. (2017). Microstructured Optical Fiber Sensors. J. Lightwave Technol..

[32] Durana G., Arrizabalaga O., Arrospide E., Aldabaldetreku G., Zubia J., Azkune M. (2017). Study of the Influence of Various Stress-Based Mechanisms on Polarization of an SM mPOF for the Development of Useful Devices. J. Lightwave Technol..

[33] Matsushige K., Radcliffe S.V., Baer E. (1976). The pressure and temperature effects on brittle-to-ductile transition in PS and PMMA. J. Appl. Polym. Sci..

[34] Sáez-Rodríguez D., Nielsen K., Bang O., Webb D.J. (2015). Simple room temperature method for polymer optical fibre cleaving. J. Lightwave Technol..

[35] Stefani A., Nielsen K., Rasmussen H.K., Bang O. (2012). Cleaving of TOPAS and PMMA microstructured polymer optical fibers: Core-shift and statistical quality optimization. Opt. Commun..

[36] Leal-Junior A.G., Frizera A., Min R., Pontes M.J., Fasano A., Woyessa G.T., Marques C. (2018). Influence of the Cladding Structure in PMMA mPOFs Mechanical Properties for Strain Sensors Applications. IEEE Sens. J..

